# Association of homocysteine with ankylosing spondylitis: a systematic review and meta-analysis

**DOI:** 10.1186/s42358-021-00175-7

**Published:** 2021-03-10

**Authors:** Hui-hui Li, Xue-quan Li, Lin-tao Sai, Yi Cui, Jia-hui Xu, Chi Zhou, Jing Zheng, Xing-fu Li, Hua-xiang Liu, Ying-jie Zhao

**Affiliations:** 1 Department of Obstetrics and Gynecology, Qilu Hospital, Cheeloo College of Medicine, Shandong University, Jinan, 250012 Shandong China; 2 Department of Obstetrics and Gynecology, University of Wisconsin-Madison, Madison, WI 53715 USA; 3 Department of Gastroenterology, Shouguang People’s Hospital, Shouguang, 262700 Shandong China; 4 Department of Infectious Diseases, Qilu Hospital, Cheeloo College of Medicine, Shandong University, Jinan, 250012 Shandong China; 5 Department of Intensive Care Unit, Qilu Hospital, Cheeloo College of Medicine, Shandong University, Jinan, 250012 Shandong China; 6 Shandong First Medical University & Shandong Academic of Medical Sciences, Jinan, 250000 Shandong China; 7 School of Animal and Comparative Biomedical Sciences, University of Arizona, Tucson, AZ USA; 8 Department of Rheumatology, Qilu Hospital, Cheeloo College of Medicine, Shandong University, Jinan, 250012 Shandong China

**Keywords:** Homocysteine, Meta-analysis, Ankylosing spondylitis, Controls

## Abstract

**Background:**

Hyperhomocysteinemia is associated with autoimmune diseases such as ankylosing spondylitis (AS), systemic lupus erythematosus (SLE), and rheumatoid arthritis (RA). Current findings regarding plasma/serum homocysteine (HCY) levels in AS patients are inconsistent. This study aims to systematically evaluate the association between circulating HCY levels and AS.

**Methods:**

Online electronic databases (PubMed, Web of Science, Embase, ScienceDirect, China National Knowledge Infrastructure (CNKI), and Wanfang data) were used to retrieve all relevant articles published up to May 7, 2020. The pooled standardized mean difference (SMD) with 95% confidence interval (CI) was calculated using the random-effect model, Stata16 software.

**Results:**

Nine articles containing 778 AS patients and 522 controls were included in this meta-analysis. No significant differences in HCY levels were found between AS and control groups (pooled SMD = 0.46, 95% CI = − 0.30 to 1.23, *P* = 0.23). However, subgroup analysis suggested that HCY levels were significantly higher (*P* < 0.05) in the AS group treated with methotrexate (MTX) compared with the control group. In contrast, HCY levels were significantly (*P* < 0.05) lower in the AS group receiving anti-TNF-α treatment compared with the control group. No significant differences were detected between HCY levels and disease activity scores (Bath AS disease activity index, BASDAI), and methylenetetrahydrofolate reductase (MTHFR) C677T genotype.

**Conclusion:**

This meta-analysis indicates that HCY levels are similar between AS and controls, and do not correlate with disease activity. However, different medical treatments cause fluctuations of circulating HCY levels in AS patients. Further and larger-scale studies are needed to confirm these findings.

**Trial registration:**

This study was registered at international prospective register of systematic reviews (PROSPERO), registration number: CRD42020184426.

## Background

Ankylosing spondylitis (AS) is an immune-associated systemic inflammatory rheumatic disease characterized primarily by progressive inflammation of the spine, sacroiliitis, and various extra-articular manifestations such as anterior uveitis, inflammatory bowel disease, subclinical inflammation of the gut, psoriasis, airway disease, and interstitial lung abnormalities [1–6]. Increasing evidence indicates that AS increases cardiovascular morbidity and mortality as compared with the general population [7].

Homocysteine (HCY) is a sulfur-containing amino acid formed during the metabolism of methionine to cysteine [8]. HCY levels are elevated in many acquired disorders such as cardiovascular disease, cerebrovascular disease, dementia-type disorders, osteoporosis-associated fractures, chronic renal disease, autoimmune diseases, inflammatory diseases, deficiency of vitamins B6, B12, or folic acid, as well as during anti-folate drug therapy [9–19] Elevated HCY may trigger autoimmune reactions through binding and structurally modifying specific proteins, resulting in the formation of neoantigens that are potentially relevant either in the onset of specific autoimmune diseases or in the progression of the associated cardiovascular damage [20].

HCY could modify Human Leukocyte Antigens-B27 (HLA-B27) through forming a disulfide bond with an unpaired cysteine residue at position 67 (Cys67) of the HLA-B27 heavy chain [21], and subsequently, be destroyed by the abnormal autoimmunological reactions [21]. An in vitro study has shown HCY could induce specific cytotoxic T lymphocytes (CTLs), and HLA-B27-restricted HCY-specific CTLs are more often found in B27-positive patients [21]. HCY-treated B cells can be specifically lysed by CTLs in patients with AS and reactive arthritis (ReA) [21]. Evidence also suggests that Salmonella infection could lead to the modification of HLA antigens and such modified HLA antigens can be recognized by HCY-specific CTLs [21]. Collectively, these findings support the notion that HCY may be involved in the mechanism underlying HLA-B27-associated AS.

Previous studies have shown both pro-inflammatory and anti-inflammatory properties of HCY [22–25]. An association between hyperhomocysteinemia and inflammation has been identified in human and experimental animal models [17, 26]. In vitro, HCY induces mRNA and protein expression of the inflammatory cytokines including tumor necrosis factor (TNF)-α, Interleukin (IL)-1β, IL-6, IL-8, and IL-12 in human monocytes [27]. Correlations of HCY with soluble 75-kDa TNF-receptor (sTNF-R75) has also been found in RA patients [28]. HCY may activate nuclear factor-kappaB (NF-κB) activation [29], which may lead to increased chemokine expression in vascular smooth muscle cells (VSMCs) and macrophages [30, 31]. HCY, at the range of physiologic concentrations, enhances monocyte proliferation in vitro [27]. HCY may also be involved in corona virus disease-19 (COVID-19) infection via transsulfuration pathway [32], which is catalyzed by cystathionine β-synthase and cystathionine γ-lyase (CSE) and serves as a modulator of inflammation [33]. Nevertheless, HCY significantly ameliorates cholesterol-induced inflammation in an in vivo hypercholesterolemic rat model possibly by acting on the tissue plasminogen activator (tPA)-induced process [24].

Based on the facts that AS is associated with increased cardiovascular morbidity and mortality, the high level of HCY is considered a well-known risk factor for cardiovascular disease [7, 8], it is necessary to define the association between HCY and AS. However, the conclusions of HCY levels reported in AS patients are inconsistent. Several studies reported high levels of serum/plasma HCY levels in AS patients [34–38]. In contrast, others claimed that HCY levels in AS patients were similar or lower compared with control patients [16, 39–41]. Therefore, in this meta-analysis, we aim to determine HCY levels in AS and investigate the correlation between HCY levels and disease activity and medical treatments.

## Methods

### Search strategy

We performed electronic literature searches in PubMed, Web of Science, Embase, ScienceDirect, China National Knowledge Infrastructure (CNKI), and Wanfang data up to May 7, 2020 with keywords including Homocysteine, Hyperhomocysteinemia, Ankylosing, and Spondylitis.

### Inclusion criteria and exclusion criteria

**Fig. 1:**
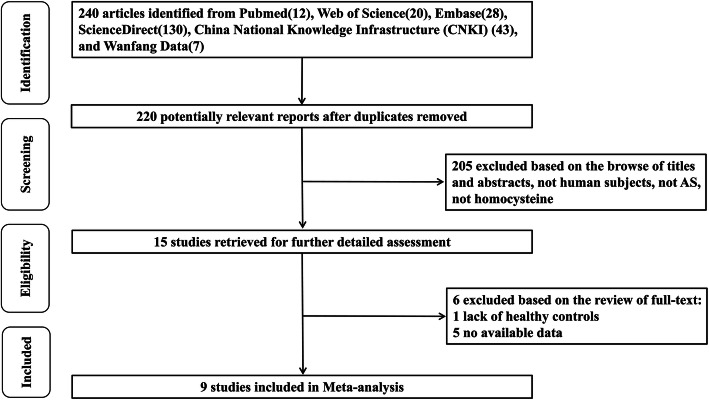
Flow chart of selected articles

The article inclusion criteria were as follows: (1) a case-control or cross-sectional study; (2) based on adults; (3) AS patients must conform to the American College of Rheumatology (ACR) criteria [42] or European Spondyloarthropathy Study Group (ESSG) criteria for classification of spondylarthropathies [43]; and (4) serum or plasma HCY concentrations of both patients and controls were available. Articles were not limited to geographic location or language of publication. Articles excluded in this meta-analysis were as follows: (1) reviews; (2) animal studies; and (3) lack of controls. A detailed flow chart of article inclusion and exclusion process was presented in Fig. 1. All articles obtained electronically were examined independently by two researchers.

### Data extraction and quality assessment

Each of the enrolled articles was extracted by two researchers independently for the following information: first author’s name, year of publication, study design, the language of the article, region, participants’ characteristics (age and gender), disease activity score (Bath AS disease activity index, BASDAI) [44], disease duration, mean ± SD or mean ± SEM of the plasma/serum HCY levels in AS and control groups, measurement method, sample type, and any other relevant information. Meta-analysis was performed according to the Preferred Reporting Items for Systematic Reviews and Meta-Analysis (PRISMA) guidelines [45]. Any disagreement on study inclusion or data extraction was discussed with a third reviewer to reach a consensus. The Newcastle-Ottawa quality assessment scale (NOS) was used to assess the methodological quality of eligible studies [46].

### Statistical analysis

The extracted results that were presented in median, standard error, range, and interquartile range were converted to mean and standard deviation (SD) [47, 48]. The mean and SD (mean ± SD) were extracted or estimated in each study. For each study, the standardized mean difference (SMD) and 95% confidence intervals (95% CI) were elaborately shown by the forest plot. Cochrane *Q* statistics (chi-square test, χ2) were employed to evaluate statistical heterogeneity. The *I*-square (*I*^2^) statistics was used to further assess the extent of heterogeneity (*I*^2^ = ([*Q − df*]) × 100%) [49]. Based on the Cochrane Handbook for Systematic Reviews’ recommendations, we interpreted *I*^2^ statistic between 0 and 40% as representing an insignificant amount of heterogeneity; 30 to 60% as moderate heterogeneity; 50 to 90% as substantial heterogeneity; and 75 to 100% as considerable heterogeneity [50]. When *P* < 0.05 for the *Q* test or *I*^2^ > 50, it indicated that heterogeneity was significant in the study. Assuming that the heterogeneity is significant, the random-effects model was adopted to pool the SMD value; or else, the fixed-effect model was adopted. Subgroup analysis was performed to discover the sources of heterogeneity. Sensitivity analysis was also executed to detect studies that extremely contributed to the observed heterogeneity. This was done by omitting each study one after the other to measure its impact on the summary estimate. To evaluate the publication bias, Egger’s and Begg’s tests were used. Stata 16 software was used in this meta-analysis to perform statistical analysis.

## Results

### Publication search and study characteristics

Out of a total of 15 relevant studies found, 9 original studies met our inclusion criteria and were selected for this meta-analysis. These 9 studies were published between 2005 and 2020 and included 778 AS patients and 522 controls [16, 34–41]. Of the 9 studies, 4 were conducted in Asia, 3 in Asia-Europe (Turkey), 1 in Europe, and 1 in South America. As of the controls, 1 study included non-inflammatory low back pain [39], 1 included osteoarthritis or soft tissue rheumatism [41], and the other 7 included healthy controls [16, 34–38, 40]. Our methodological quality assessment showed the NOS scores of included studies ranged from 7 to 8. The characteristics of the included studies are summarized in Table 1. Extracted data on circulating HCY levels were presented in Table 2.

**Table 1:** Characteristics of individual studies included

First author, year	Region	AS					Control						
		Age (year) (mean ± sd)	BMI	Sex ratio (M/F)	BASDAI	Duration (year) (mean ± sd)	Age (year) (mean ± sd)	BMI	Sex ratio (M/F)	Assay method, sample	Study type	Criteria for the classification	NOS
Shu JL, 2020 [16]	Mainland China	41.6 ± 8.6	NA	30/5	NA	NA	39.7 ± 14.4	NA	36/5	NA	CS	ACR	7
Geçene M, 2013 [39]	Turkey	36.7 ± 4.8	24.9 ± 3.1	50/0	2.4 ± 1.7	NA	36.3 ± 4.7	25.0 ± 3.0	50/0	HPLC, plasma	CS	ACR	7
Mao N, 2012 [34]	Mainland China	NA	NA	NA	NA	NA	NA	NA	NA	ELISA, plasma	CS	ACR	7
Capkin E, 2012 [40]	Turkey	36.4 ± 11.2	NA	88/20	3.7 ± 1.6	8.4 ± 5.6	38.2 ± 13.0	NA	49/16	ELISA, plasma	CS	ACR	7
Başkan BM, 2009 [35]	Turkey	40.1 ± 11.0	NA	NA	4.2 ± 1.7	10.0 ± 7.7	38.1 ± 0.6	NA	NA	NA	CS	ACR	7
Gonzalez-Lopez L, 2008 [36]	Mexico	39.9 ± 8.4	26.0 ± 2.8	73/24	5.4 ± 2.1	7.8 ± 6.0	38.9 ± 7.8	25.8 ± 4.2	73/24	FPIA, Serum	CSS	ACR	8
Wei JC, 2007 [37]	Taiwan, China	NA	NA	NA	NA	NA	NA	NA	NA	ELISA, plasma	CSS	ACR	7
Malesci D, 2007 [41]	Italy	47.6 ± 11.8	29.5 ± 4.4	21/3	2.7 ± 1.8	18.5 ± 10.8	49.6 ± 6.0	NA	16/3	NA	CS	ACR&ESSG	8
Xu XY, 2005 [38]	Mainland China	32.1 ± 13.7	NA	44/16	NA	NA	30.8 ± 19.7	NA	30/32	FPIA, plasma	CS	ACR	7

*AS* Ankylosing spondylitis, *BMI* Body mass index, *BASDAI* Bath AS disease activity index, *NOS* Newcastle-Ottawa Scale, *NA* Not available, *HPLC* High performance liquid chromatography, *ELISA* Enzyme-linked immunosorbent assays, *FPIA* Fluorescence polarization immunoassay, *CS* Case control, *CSS* Cross-sectional study, *ACR* American College of Rheumatology, *ESSG* European Spondyloarthropathy Study Group

**Table 2:** Extracted data on HCY levels of 9 studies included

First author, year	AS (μmol/L)			Control (μmol/L)		
	*N*	Mean	SD	*N*	Mean	SD
Shu JL, 2020 [16]	35	12.27	7.52	41	13.94	3.73
Geçene M, 2013 [39]	50	14.26	9.96	50	11.81	5.53
Mao N, 2012 [34]	200	18.71	2.42	120	10.97	2.93
Capkin E, 2012 [40]	108	18.90	8.70	65	23.80	5.80
Başkan BM, 2009 [35]	92	17.18	12.47	58	13.16	4.09
Gonzalez-Lopez L, 2008 [35]	97	11.35	5.39	97	9.61	2.33
Wei JC, 2007 [37]	112	9.91	3.47	10	8.60	1.20
Malesci D, 2007 [41]	24	8.70	2.00	19	9.10	1.20
Xu XY, 2005 [38]	60	16.47	6.50	62	12.24	3.58

*HCY* Homocysteine, *N* Number, *SD* Standard deviation

### Results of meta-analysis

**Fig. 2:**
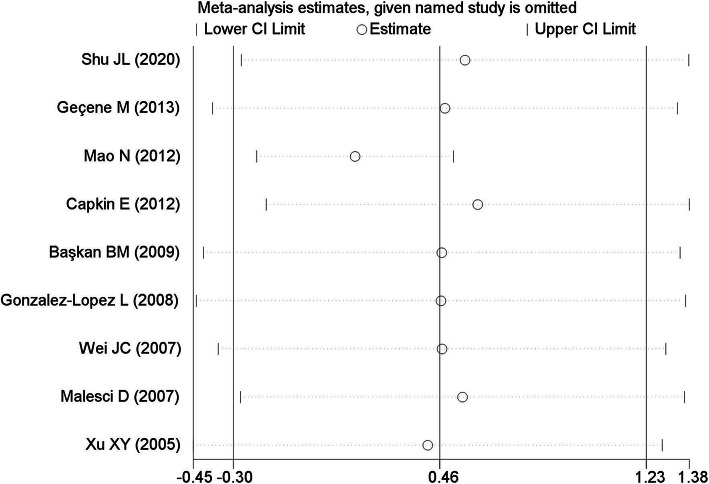
Sensitivity analysis of the included studies. The two vertical axes, vertical middle axis, hollow circles, and two ends of the dotted lines respectively represent 95% confidence interval, overall odd ratio, pooled odd ratios, and 95% confidence interval

Publication bias was assessed by Egger’s regression test (*t* = − 0.74, *P* = 0.48) and Begg’s test (*z* = − 0.31, *P* = 1.25), suggesting no publication bias present. Among the enrolled studies, significant heterogeneity was observed (*I*^2^ = 97.3%, *P* < 0.01). Sensitivity analysis by sequentially omitting individual studies did not significantly change the pooled results, suggesting that these results were stable (Fig. 2).

### HCY levels and subgroup analysis

**Fig. 3:**
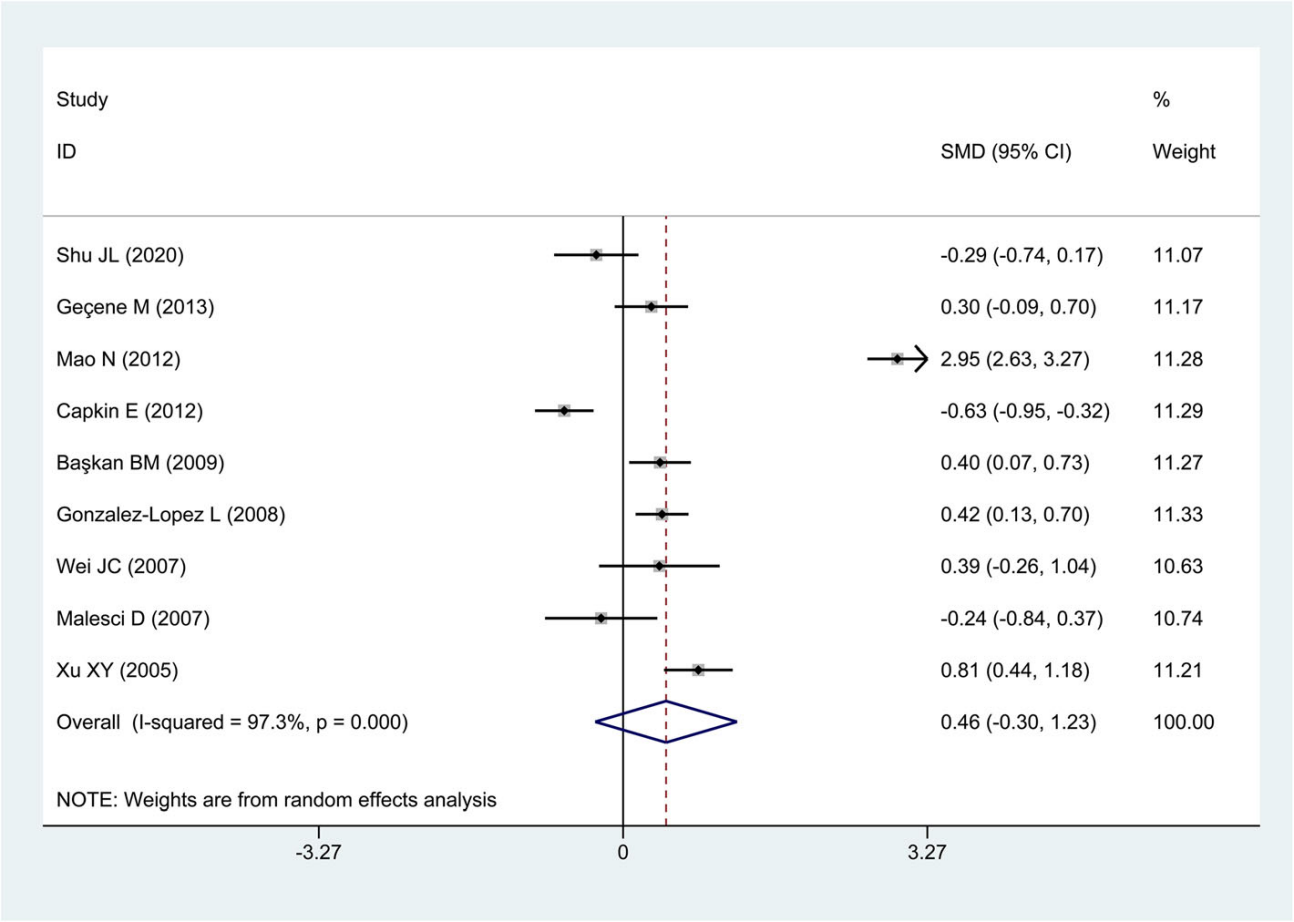
Forest plot of 9 studies in HCY levels for AS patients versus controls, based on random effects model

The random-effect model was used for the analysis of results in this study based on the result that significant heterogeneity was observed among the enrolled studies (*P* < 0.01). There were no significant differences in circulating HCY levels between AS patients and controls (pooled SMD = 0.46, 95% CI = − 0.30 to 1.23, *P* = 0.23) (Fig. 3). Subgroup analysis stratified by region, sample size, disease activity, smoker or not, methylenetetrahydrofolate reductase (MTHFR) C677T genotype, and medication treatment were performed (Table 3). As of disease activity, 1 study defined that patients with BASDAI values ≥4 were regarded as active AS group [40]. Two studies defined that patients with BASDAI values > 3 and ESR > 20 mm/h were regarded as active AS group [35, 37]. To conduct medication treatment subgroup analysis, we analyzed four subgroups: nonsteroidal anti-inflammatory drugs (NSAIDs), sulfasalazine (SSZ), SSZ + methotrexate (MTX), and anti-TNF-α. In SSZ + MTX treatment subgroup, HCY levels in AS patients were significantly higher than those in controls (pooled SMD = 0.915, 95% CI = 0.312 to 1.518, *P* = 0.003). In anti-TNF-α treatment subgroup, HCY levels in AS patients were significantly lower than in controls (pooled SMD = − 0.774, 95% CI = − 1.163 to − 0.385, *P* < 0.001). Meanwhile, no significant differences of HCY levels between AS patients and controls were detected in NSAIDs or SSZ treatment subgroup. In particular, no significant correlation between HCY levels and disease activity, and MTHFR C677T genotype, as well as in region, sample size, and smoking subgroups was detected.

**Table 3:** Subgroup analysis of HCY levels in AS

Subgroups		*N*	SMD (95% CI)	*Z*	*P*	Heterogeneity test	
						*P*	*I*^*2*^ *(%)*
Region	Asia	4	0.974 (−0.573, 2.521)	1.23	0.217	< 0.001	98.1%
	Asia + Europe (Turkey)	3	0.018 (− 0.661, 0.698)	0.05	0.958	< 0.001	91.4%
	Europe/America	2	0.148 (−0.484, 0.780)	0.46	0.646	0.055	73.0%
	Combined	9	0.463 (−0.300, 1.226)	1.19	0.234	< 0.001	97.3%
Sample size	≥ 50	6	0.708 (−0.283, 1.700)	1.40	0.162	< 0.001	98.1%
	< 50	2	−0.269 (− 0.632, 0.093)	1.46	0.145	0.891	0.0%
	Combined	8	0.471 (−0.355, 1.298)	1.12	0.264	< 0.001	97.6%
Disease activity	active	3	0.048 (−0.904, 1.000)	0.10	0.921	< 0.001	92.0%
	inactive	3	0.078 (−0.733, 0.889)	0.19	0.851	< 0.001	89.0%
	Combined	6	0.059 (−0.488, 0.606)	0.21	0.833	< 0.001	88.5%
Smoking	Smoker	1	0.497 (−0.066, 1.060)	1.73	0.084	NA	NA
	Non-smoker	1	0.201 (−0.355, 0.757)	0.71	0.478	NA	NA
	Combined	2	0.347 (−0.048, 0.743)	1.72	0.085	0.464	0.0%
MTHFR C677T genotype	CC	2	1.599 (−0.864, 4.062)	1.27	0.203	< 0.001	98.1%
	CT	2	1.693(−1.256, 4.642)	1.13	0.260	< 0.001	97.7%
	TT	2	2.771(−1.141, 6.682)	1.39	0.165	< 0.001	92.2%
	Combined	6	1.992 (0.663, 3.320)	2.94	0.003	< 0.001	95.7%
Treatment	NSAIDs	2	−0.365(− 1.020, 0.291)	1.09	0.275	0.092	64.8%
	SSZ	2	0.024 (−0.872, 0.920)	0.05	0.958	0.003	88.7%
	SSZ + MTX	1	0.915 (0.312, 1.518)	2.97	0.003	NA	NA
	Anti-TNF-α	1	−0.774 (−1.163, − 0.385)	3.90	< 0.001	NA	NA
	Combined	6	−0.094 (− 0.634, 0.445)	0.34	0.732	0.000	87.7%

*HCY* Homocysteine, *NA* Not available, *MTHFR* Methylenetetrahydrofolate reductase, *NSAIDs* Non-steroidal anti-inflammatory drugs, *SSZ* Sulfasalazine, *MTX* Methotrexate, *TNF* Tumor necrosis factor

## Discussion

A positive relationship was reported between circulating HCY levels and certain clinical features of rheumatoid arthritis (RA), such as higher disease activity [51] and higher radiological damage [52]. A significant correlation also exists between HCY levels and the various indexes of disease activity of systemic lupus erythematosus (SLE), such as erythrocyte sedimentation rate, anti-double-stranded DNA, complement levels in patients of SLE [15]. Intensive steroid therapy in RA patients results in significant HCY reduction [53]. In contrast, Shu et al. has reported that HCY levels are not associated with C-reactive protein (CRP), erythrocyte sedimentation rate (ESR), rheumatoid factor (RF), and anti-citrullinated protein antibody (ACPA) in RA [16]. Our study indicates no significant correlation between HCY levels and AS disease activity. However, subgroup analysis suggests that elevated HCY levels are associated with the AS group treated with MTX. This association may be attributed to the inhibitory effect of MTX on the levels of folate [19, 40]. Specifically, MTX treatment influences folate metabolism in RA patients, leading to a decrease in serum folate levels and a rise in HCY levels [54]. However, while MTX increases plasma HCY levels, folate supplementation decreases HCY concentrations, protecting against potential cardiovascular risks in RA patients [55]. In clinical practice, folate supplement is used to reduce side-effect incidence of MTX in RA patients, suggesting that folate supplement should be considered for AS patients treated with MTX. Of the 9 studies included in this analysis, 4 recruited AS patients on MTX [35–37, 41], 2 of which used folate supplementation [35, 36]. Of the other 5 studies, 3 didn’t use MTX [38–40] and 2 didn’t mention any treatment method [16, 34].

SSZ has also been reported to have anti-folate properties [56], implying SSZ may increase HCY levels in patients. This is supported by the observation that plasma HCY levels was increased significantly in AS patients under SSZ, and SSZ/MTX combination treatment [37]. However, our subgroup analysis showed no significant differences in HCY levels in AS patients treated with SSZ compared with controls. In addition, HCY levels were significantly lower in AS group treated with anti-TNF-α regimen. These findings support the note that different medical treatments may influence HCY levels. However, the reasons attributed to the above results remain unknown. In this meta-analysis, two studies contain HCY levels and treatment methods in AS patients (*n* = 198) [35, 40]. The above findings might be a result of the small sample size employed in the individual studies. Further studies are needed to confirm these findings.

HCY levels are known to be controlled by methylenetetrahydrofolate reductase (MTHFR), which is a key enzyme in HCY metabolism [57]. MTHFR C677T polymorphism causes a thermolability of MTHFR, reducing its enzymatic activity [57]. Such a reduction inhibits the formation of 5-methyltetrahydrofolate, which serves as a methyl donor during the remethylation of HCY to methionine [57]. Studies indicated that the TT genotype of the MTHFR C677T polymorphism exhibits higher plasma HCY concentrations than CT heterozygotes and CC homozygotes [58, 59]. Besides, Mao et al. reported that high plasma HCY levels are associated with MTHFR 677TT polymorphism as compared with the CC or CT genotype in AS patients [34]. In this meta-analysis, subgroup analysis showed no significant correlation between all 3 genotypes of the MTHFR C677T polymorphism and HCY levels in AS. This result agrees with the previous report which showed no association between MTHFR 677TT polymorphism and plasma HCY levels in obese children and adolescents [60] and no statistically significant differences according to the frequency of MTHFR C677T polymorphism between AS patients and controls [39].

As a well-known risk factor for cardiovascular disease, HCY can mediate the development of the cardiovascular disease by acting adversely on vascular endothelium and smooth muscle cells [61]. Elevated HCY levels may also enhance oxidative stress and inflammation of vascular endothelial cells and reduce the production and bioavailability of nitric oxide (a strong relaxing factor) by endothelium [62]. HCY stimulates the proliferation of vascular smooth muscle cells, synthesis of collagen, and deterioration of arterial wall elastic material [61]. AS also significantly increases risks of myocardial infarction and stroke [63]. Multiple factors contribute to such increased cardiovascular risk. In this regard, systemic inflammation and high disease activity play pivotal roles in the process [64]. Another possible factor is the proatherogenic profile of AS patients who were smokers and/or hypertensive with a poor atherogenic lipid profile [64]. Recommendations from EULAR pointed out that disease activity should be controlled optimally to lower CVD risk in all patients with AS, RA or psoriatic arthritis (PsA) [65]. In addition, MTX treatment decreases the acute myocardial infarction (AMI) among RA patients [66], suggesting that MTX plays a role in protecting patients from CVD risk, possibly via controlling the inflammatory process although MTX elevates HCY levels.

To our knowledge, this is the first meta-analysis that provides evidence of HCY levels in AS patients compared with controls. We are able to extract valid and accurate results from the individual articles and use a sample size of 778 patients of AS and 522 of controls for HCY levels to increase the statistical power and resolution of our analysis compared with the individual studies. However, several limitations are recognized. First, the significant heterogeneity in our meta-analysis may restrict the generalization of the pooled result. Second, due to the lack of sufficient data to perform more exhaustive subgroup analysis and meta-regression, the origin of heterogeneity could not be fully revealed.

## Conclusions

This meta-analysis suggests that HCY may partially participate in the pathogenesis of AS, although it is not a major contributing factor. Further and larger studies are needed to confirm these findings.

## Data Availability

The datasets used and/or analyzed during the current study are available from the corresponding author on reasonable request.
